# Number of standard modifiable risk factors and mortality in patients with first-presentation ST-segment elevation myocardial infarction: insights from China Acute Myocardial Infarction registry

**DOI:** 10.1186/s12916-022-02418-w

**Published:** 2022-07-06

**Authors:** Sidong Li, Xiaojin Gao, Jingang Yang, Haiyan Xu, Yang Wang, Yanyan Zhao, Lu Yin, Chao Wu, Yi Wang, Yang Zheng, Bao Li, Xuan Zhang, Yunqing Ye, Rui Fu, Qiuting Dong, Hui Sun, Xinxin Yan, Yuan Wu, Jun Zhang, Chen Jin, Wei Li, Yuejin Yang, on behalf of CAMI investigators

**Affiliations:** 1 Medical Research and Biometrics Center, State Key Laboratory of Cardiovascular Disease, Fuwai Hospital, National Center for Cardiovascular Diseases, Chinese Academy of Medical Sciences and Peking Union Medical College, Beijing, 102300 China; 2 Coronary Heart Disease Center, Department of Cardiology, Fuwai Hospital, National Center for Cardiovascular Diseases, Chinese Academy of Medical Science and Peking Union Medical College, 167 Beilishi Rd, Beijing, 100037 China; 3 Xiamen Cardiovascular Hospital, Xiamen, Fujian China; 4 The First Hospital of Jilin University, Jilin, China; 5 The Affiliated Cardiovascular Hospital of Shanxi Medical University, Taiyuan, Shanxi China

**Keywords:** Modifiable risk factors, ST-elevation myocardial infarction, Mortality, China

## Abstract

**Background:**

Recent publications reported a paradoxical finding that there was an inverse association between the number of standard modifiable risk factors (SMuRFs; smoking, hypertension, diabetes, and hyperlipidemia) and mortality in patients with myocardial infarction. However, the current evidence is only limited to those highly developed countries with advanced medical management systems.

**Methods:**

The China Acute Myocardial Infarction registry is a prospective observational study including patients with acute myocardial infarction from three-level hospitals across 31 administrative regions throughout mainland China. A total of 16,228 patients with first-presentation ST-elevation myocardial infarction (STEMI) admitted to hospitals from January 2013 to September 2014 were enrolled in the current analysis. Cox proportional hazard models adjusting for baseline characteristics, clinical profiles at presentation, and in-hospital treatments were used to assess the association of the number of SMuRFs with all-cause mortality at 30 days after STEMI presentation.

**Results:**

A total of 1918 (11.8%), 11,503 (70.9%), and 2807 (17.3%) patients had 0, 1–2, and 3–4 SMuRFs at presentation, respectively. Patients with fewer SMuRFs were older and more likely to be females, experienced longer pre-hospital delays, and were less likely to receive primary percutaneous coronary intervention and evidence-based medications. Compared with those without any SMuRF, patients with 1–2 SMuRFs and 3–4 SMuRFs were associated with an HR of 0.74 (95% CI, 0.63–0.87) and 0.63 (0.51–0.77) for all-cause mortality up to 30 days in the unadjusted model (*P*_trend_ < 0.0001). However, after multivariate adjustment, the number of SMuRFs was positively associated with increased mortality risk (HR for 1–2 SMuRFs, 1.15 [0.95–1.39]; HR for 3–4 SMuRFs, 1.31 [1.02–1.68]; *P*_trend_ = 0.03), and the association was only significant among patients admitted to hospitals beyond 12 h from onset (HR for 1–2 SMuRFs, 1.39 [1.03–1.87]; HR for 3–4 SMuRFs, 2.06 [1.41–3.01]) but not their counterparts (*P*_interaction_ = 0.01).

**Conclusions:**

The increased crude mortality risk among patients without SMuRFs is explained by confounding factors related to their poor risk profiles (old age, longer pre-hospital delays, and poor clinical management). After multivariate adjustment, a higher risk-factor burden was associated with poor prognosis among patients with STEMI.

**Supplementary Information:**

The online version contains supplementary material available at 10.1186/s12916-022-02418-w.

## Background

The importance of early identification and target interventions against standard modifiable risk factors (SMuRFs; smoking, hypertension, diabetes, and hyperlipidemia) in the development and progression of coronary artery diseases (CAD) has been well recognized [1–3]. However, there is a substantial proportion of patients presenting acute myocardial infarction in the absence of these risk factors, estimated to be approximately 29.6 million cases per year [4–6]. Nonetheless, this group of patients is easily overlooked in clinical trials and guidelines and there is a lack of best approach to managing them [4].

Crucially, recent publications have reported a paradoxical finding that there was an inverse association between the number of risk factors and mortality in patients with CAD [7–10]. Some studies hypothesized that this finding could be explained by residual confounding caused by older age and other unfavorable clinical characteristics in patients without fewer SMuRFs, while the association persisted after multivariate adjustment [10, 11]. Most recently, a large-scale registry from Sweden demonstrated that the increased mortality among patients with first-presentation ST-elevation myocardial infarction (STEMI) in the absence of any SMuRF could be explained by the suboptimal prescription rates of evidence-based pharmacotherapy [7]. However, data documenting the clinical outcomes of patients without any SMuRFs are still limited and the underlying mechanisms for the increased mortality rate among this group of patients are not clear. Moreover, current evidence is predominantly from highly developed countries, where patients can homogeneously receive high-quality emergency medical service and standardized evidence-based clinical management [7–14], whereas, in China, a large proportion of patients might experience long pre-hospital delays and suboptimal medical care, especially in those low-level hospitals and remote areas [15]. A recent study even reported that the rate of reperfusion eligibility decreased among patients with STEMI from 2011 to 2015 in China, which was majorly driven by the increased prevalence of long pre-hospital delay [16]. Thus, it is important to evaluate the clinical profiles and outcomes of patients with different numbers of SMuRFs in such a complex setting.

The China Acute Myocardial Infarction (CAMI) registry is a prospective observational study including patients with acute myocardial infarction from three-level hospitals across 31 administrative regions throughout mainland China [17], which provides a unique opportunity to explore the association between the number of SMuRFs and clinical outcomes in a more diverse setting. In this study, we aimed at portraying and comparing the presenting characteristics, treatment, and clinical outcomes of STEMI patients with different numbers of SMuRFs.

## Methods

### Study participants

Details of the CAMI registry have been described and published elsewhere [15, 17]. To be brief, this study included three levels of hospitals (including provincial-, prefectural-, and county-level hospitals, representing typical of the Chinese vertical governmental and administrative models) covering all 31 provinces and municipalities throughout mainland China since January 2013. Within each province or municipality in mainland China, one of the largest provincial-level academic hospitals (high-level) was invited, then the local principal investigators of these hospitals recommended prefectural-level hospitals (medium-level) within their provinces and municipalities and county-level hospitals (low-level) within selected prefectures. Finally, a total of 108 hospitals (including 31 provincial-level hospitals, 45 prefectural-level hospitals, and 32 county-level hospitals) were included in the CAMI registry. These hospitals were selected to achieve broad coverage of geographical regions and comprehensively reflect the routine medical care system for patients with acute myocardial infarction (AMI) in China (Additional file 1: Fig. S1) [17]. In each participating hospital, patients with a primary diagnosis of AMI including STEMI and non-STEMI admitted within 7 days after the onset of ischemic symptoms were consecutively enrolled into the registry. Only patients with a final diagnosis meeting the third Universal Definition for Myocardial Infarction, including types 1, 2, 3, 4b, and 4c, were eligible in the CAMI study. This study was registered in clinicaltrials.gov (identifier: NCT01874691) and approved by the institutional review board central committee at Fuwai Hospital. Written informed consent was obtained from eligible patients.

Between January 2013 and September 2014, 19,112 patients with a primary diagnosis of STEMI were registered in CAMI. A total of 16,358 patients had no prior history of coronary artery disease (CAD; percutaneous coronary intervention [PCI], coronary artery bypass graft [CABG], or myocardial infarction). After further excluding patients with incomplete information on any one of SMuRFs, a total of 16,228 patients with first-presentation STEMI were included in the main analysis (Additional file 1: Fig. S2).

### Data collection

Enrolment procedures, data collection, and follow-up visits were all conducted by trained physicians at each participating site using predefined, standardized, and unified definitions. Comprehensive data (including demographic characteristics, medical history, pre-hospital medical contact, presenting characteristics, biochemical and electrocardiographic findings, treatment practices, and in-hospital and follow-up outcome events) was collected using systematic data entry and transmission procedures with rigorous data quality control [15, 17]. The definitions of the study variables are listed in Additional file 1: Table S1.

Similar to previous studies, we focused on four conventional risk factors including current smoking, hypertension, hyperlipidemia, and diabetes [1, 7, 18]. Current smoking was defined as smoking regularly within the past month before admission. Hypertension was defined as self-reported hypertension or using antihypertensive medications before admission. Hyperlipidemia was defined as self-reported hyperlipidemia, using lipid-lowering medications before admission, an LDL-C concentration of 3.37 mmol/L or higher, or a total cholesterol concentration of 5.18 mmol/L or higher during hospitalization [19]. Diabetes was defined as self-reported diabetes, a glucose concentration of 11.1 mmol/L or higher, or a glycated haemoglobinA1c of 6.5% or higher [20]. All these laboratory indexes referred to the first measurements after admission. Similar to prior literature, we categorized patients into three groups according to the number of SMuRFs: 0, 1–2, and 3–4 SMuRFs [8]. These groups were selected to avoid a small sample size within groups and to achieve the possibility of evaluating trends based on the number of SMuRFs. Baseline information stratified by the number of SMuRFs is also listed in Additional file 1: Table S2.

### Outcomes

Follow-up information was collected by clinical investigators at the clinic visit or by telephone call (at 30 days and 6, 12, 18, and 24 months) and re-checked by an independent team. For patients who cannot be successfully contacted in follow-up visits, survival status was identified through linkages to the national registered residence system using ID numbers. Standardized definitions of outcome events have been published previously [15]. The primary outcome in this study was 30-day mortality after STEMI, and the secondary outcome is in-hospital mortality and 2-year mortality after STEMI.

### Statistical analysis

Demographic characteristics, clinical profiles, treatments, and the rates of in-hospital outcomes were compared among different groups. Continuous variables were described by mean (SD) or median (interquartile range) and were compared by analysis of variance or the Kruskal-Wallis *H* test as appropriate. Categorical variables were described as frequencies and percentages, and comparison was performed with the chi-square test or Fisher’s exact test. Thirty-day and 2-year mortality were demonstrated by Kaplan-Meier curves. For 2-year mortality, we also conducted a Landmark analysis using 30 days as a cut-off point to evaluate the different effects of the number of SMuRFs on both short-term and long-term mortality.

The association between the number of SMuRFs and the primary outcome (30-day mortality) was assessed with Cox proportional hazards models. This study was a post hoc analysis, and covariates were selected based on prior literature and clinical relevance, and we also included some covariates which were differentially distributed between groups and have been identified to be significantly associated with clinical outcomes in existing evidence. To better clarify the drivers of the mortality differences across groups, we used five models sequentially adjusting for a series of clinically relevant and coherent factors: (1) unadjusted; (2) adjusted for age only; (3) adjusted for age, sex, education, hospital levels, BMI, family history of CAD, and prior medical history (stroke and prior chronic obstructive pulmonary disease [COPD]), and pre-admission aspirin; (4) further adjusting for presenting characteristics (onset-to-arrival time, pre-admission cardiac arrest, heart rate, systolic blood pressure, plasma creatinine, Killip class, and anterior myocardial infarction); (5) additionally adjusted for in-hospital medical treatment including reperfusion strategies (primary PCI, fibrinolysis, and no reperfusion) and evidence-based medications (aspirin, P2Y12-receptor inhibitors, statin, angiotensin-converting enzyme inhibitors [ACEIs]/angiotensin receptor blockers [ARB], and β-blockers). For all these models, we conducted analyses among participants with complete data on covariates given that the proportion of missing values was very low for all included covariates (Additional file 1: Table S1). We also conducted several exploratory analyses stratified by age (<55, 55–<75, ≥75 years), sex, hospital levels (province-level, prefecture-level, and county-level), onset-to-arrival time (<12 h and ≥12 h), and reperfusion therapy (primary PCI or not). Considering the potential that patients with more severe presentation might provide less complete and reliable information on the history on risk factors [10], we also conducted an analysis stratified by Killip class (I/II and III/IV), LVEF (<40% and ≥40%), and GRACE score (≤140 and >140). In addition, we repeated analyses by excluding participants who died within the first 24 h after admission or patients transferring out to avoid the possibility of bias from incomplete data and reverse causation. Moreover, to address the influence of undiagnosed risk factors, we conducted a sensitivity analysis by removing participants without complete data on lipid measurements and glucose concentration. All analyses were done with SAS software (version 9.4). A two-sided *P* value of less than 0.05 was considered statistically significant.

## Results

A total of 16,228 patients with first-presentation STEMI were included in the current analysis. Among the overall sample, 1918 (11.8%) patients had no SMuRF at hospitalization, and 11,503 (70.9%) and 2807 (17.3%) had 1–2 and 3–4 SMuRFs at presentation, respectively. The baseline characteristics of the study population are presented in Table 1. Patients with 3–4 SMuRFs were 5 years younger than those without any SMuRF at the presentation of STEMI (3–4 SMuRFs versus no SMuRF, 59.3±11.5 versus 64.9±13.0 years, *P* < 0.0001) and more likely to be males (80.7% versus 68.7%, *P* < 0.0001). Patients with lower education levels and admitted to county-level hospitals had a lower number of SMuRFs at the first presentation of STEMI. The most common SMuRF was hypertension (48.6%), followed by current smoking (47.5%), hyperlipidemia (37.4%), and diabetes (26.6%). Obesity was highly correlated with the number of SMuRFs, and patients with a greater number of SMuRFs had a higher body mass index (*P* < 0.0001). With an increasing number of SMuRFs, patients were more likely to have a family history of CAD and a prior history of stroke, but were less common to have chronic obstructive pulmonary disease.

**Table 1:** Baseline demographic and clinical characteristics of patients with first ST-segment elevation myocardial infarction by number of cardiovascular risk factors

Variables	Total (***N*** = 16228)	No SMuRF (***N*** = 1918)	1–2 SMuRFs (***N*** = 11503)	3–4 SMuRFs (***N*** = 2807)	***P*** value
**Admission characteristics**					
Age	61.7 ± 12.5	64.9 ± 13.0	61.8 ± 12.6	59.3 ± 11.5	<0.0001
<55	4834 (30.0)	436 (22.9)	3387 (29.7)	1011 (36.2)	
55–74	8659 (53.8)	1004 (52.6)	6151 (53.9)	1504 (53.9)	
≥75	2614 (16.2)	467 (24.5)	1873 (16.4)	274 (9.8)	
Male	12381 (76.3)	1318 (68.7)	8799 (76.5)	2264 (80.7)	<0.0001
Education					<0.0001
Illiterate	1293 (8.0)	232 (12.1)	953 (8.3)	108 (3.8)	
Primary or secondary	9073 (55.9)	1052 (54.8)	6471 (56.3)	1550 (55.2)	
High	1348 (8.3)	112 (5.8)	955 (8.3)	281 (10.0)	
Unknown	4514 (27.8)	522 (27.2)	3124 (27.2)	868 (30.9)	
Hospital class					<0.0001
Province-level	5256 (32.4)	496 (25.9)	3699 (32.2)	1061 (37.8)	
Prefecture-level	8808 (54.3)	1098 (57.2)	6275 (54.6)	1435 (51.1)	
County-level	2164 (13.3)	324 (16.9)	1529 (13.3)	311 (11.1)	
Smoking status					<0.0001
Never	7062 (43.5)	1582 (82.5)	4885 (42.5)	595 (21.2)	
Former	1454 (9.0)	336 (17.5)	1020 (8.9)	98 (3.5)	
Current	7712 (47.5)	0 (0)	5598 (48.7)	2114 (75.3)	
Hypertension	7887 (48.6)	0 (0)	5426 (47.2)	2461 (87.7)	<0.0001
Diabetes	4312 (26.6)	0 (0)	2392 (20.8)	1920 (68.4)	<0.0001
Hyperlipidemia	6060 (37.4)	0 (0)	3738 (32.5)	2322 (82.7)	<0.0001
Weight, kg	68.1 ± 10.9	64.4 ± 10.5	67.9 ± 10.7	71.3 ± 11.2	<0.0001
Body mass index, kg/m^2^	24.1 ± 3.1	23.2 ± 3.0	24.1 ± 3.1	25.0 ± 3.3	<0.0001
**Medical history**					
Family history of coronary artery disease	562 (3.5)	34 (1.8)	370 (3.2)	158 (5.6)	<0.0001
Heart failure	114 (0.7)	11 (0.6)	82 (0.7)	21 (0.7)	0.76
Stroke	1399 (8.6)	95 (5.0)	1022 (8.9)	282 (10.1)	<0.0001
Peripheral arterial disease	61 (0.4)	5 (0.3)	41 (0.4)	15 (0.5)	0.26
Chronic renal failure	112 (0.7)	8 (0.4)	83 (0.7)	21 (0.8)	0.30
Chronic obstructive pulmonary disease	271 (1.7)	45 (2.4)	187 (1.6)	39 (1.4)	0.03
**Pre-hospital pharmacotherapy**					
Aspirin	937 (5.8)	38 (2.0)	594 (5.2)	305 (10.9)	<0.0001
P_2_Y_12_ inhibitor	318 (2.0)	15 (0.8)	195 (1.7)	108 (3.9)	<0.0001
Statin	942 (5.9)	0 (0)	522 (4.6)	420 (15.2)	<0.0001
β-blocker	518 (3.2)	0 (0)	303 (2.7)	215 (7.8)	<0.0001
ACEI/ARB	763 (4.8)	0 (0)	470 (4.2)	293 (10.6)	<0.0001

*SMuRF* Standard modifiable cardiovascular risk factor, *ACEI* Angiotensin-converting enzyme inhibitor, *ARB* Angiotensin receptor blocker

Compared with patients without SMuRFs, those with a greater number of SMuRFs had higher rates of using the emergency medical system and were less likely to experience longer pre-hospital delays (Table 2). The median time from symptoms to first medical contact was 370 (165–1441), 330 (140–1115), and 300 (120–1005) min among patients with no SMuRF, 1–2, and 3–4 SMuRFs, respectively (*P* < 0.0001). At presentation, patients without SMuRFs had significantly higher GRACE risk scores and lower systolic blood pressure, heart rate, and creatinine. The rate of primary PCI was 43.7% among all the patients and 58.0% among patients admitted to hospitals within 12 h from onset (Table 3). Compared with no-SMuRF patients, those with more SMuRFs were more likely to receive primary PCI among all the patients (3–4 SMuRFs versus no SMuRF, 50.4% versus 38.1%, *P* < 0.0001) and among patients admitted to hospital with 12 h from onset (3–4 SMuRFs versus no SMuRF, 63.7% versus 53.1%, *P* < 0.0001). Reasons for not receiving reperfusion therapy are listed in Additional file 1: Fig. S3, and patient/family refusal and physicians thought as out-of-time-frame were slightly more prevalent among patients with fewer SMuRFs. Of note, no significant differences were observed in door-to-balloon and door-to-needle times across patients with different numbers of SMuRFs. Over 95% of all the patients were treated with aspirin, P2Y12-receptor inhibitors, and statin during hospitalization, but patients with more SMuRFs were slightly more frequently to be treated with aspirin (*P* = 0.03), P2Y12-receptor inhibitors (*P* < 0.0001), and statin (*P* = 0.006). More obvious disparities were observed for in-hospital usage of β-blocker, ACEI/ARB, heparin, and Glycoprotein IIb or IIIa inhibitor (all *P* values < 0.0001). Similar disparities were also observed for medications at discharge. Since medication use was influenced by several aspects, we also compared the proportions of evidence-based medications adjusting for baseline characteristics, clinical profiles, and reperfusion strategies (Additional file 1: Table S3). The difference in adjusted proportions of using ACEI/ARB (both in-hospital and at discharge) and statin (at discharge) was significant.

**Table 2:** Presentation characteristics of patients with first ST-segment elevation myocardial infarction by number of cardiovascular risk factors

Variables	Total (***N*** = 16228)	No SMuRF (***N*** = 1918)	1–2 SMuRFs (***N*** = 11503)	3–4 SMuRFs (***N*** = 2807)	***P*** value
**Presentation characteristics**					
Emergency medical system	1736 (10.7)	186 (9.7)	1204 (10.5)	346 (12.4)	0.0009
Onset-to-arrival time					<0.0001
<3 h	3844 (23.9)	392 (20.7)	2681 (23.5)	771 (27.6)	
3–12 h	6974 (43.3)	811 (42.9)	4965 (43.5)	1198 (42.9)	
12–24 h	1665 (10.3)	197 (10.4)	1190 (10.4)	278 (10.0)	
1–7 d	3619 (22.5)	491 (26.0)	2584 (22.6)	544 (19.5)	
Time for symptoms to first medical contact, minute	328 (139–1124)	370 (165–1441)	330 (140–1115)	300 (120–1005)	0.0001
GRACE risk score	148.4±35.4	156.1±35.7	148.5±34.9	143.1±36.5	<0.0001
Systolic blood pressure, mmHg	127.3±24.9	120.5±22.1	127.1±24.5	133.1±27.0	<0.0001
Heart rate, beats/min	77.4±18.1	76.8±18.0	77.2±18.0	78.9±18.6	<0.0001
Cardiac arrest at presentation	207 (1.3)	24 (1.3)	144 (1.3)	39 (1.4)	0.84
Killip class					0.21
I	12466 (77.2)	1484 (77.7)	8846 (77.2)	2136 (76.4)	
II	2510 (15.5)	276 (14.5)	1805 (15.8)	429 (15.3)	
III	566 (3.5)	68 (3.6)	385 (3.4)	113 (4.0)	
IV	615 (3.8)	81 (4.2)	416 (3.6)	118 (4.2)	
LVEF					0.06
Normal, >50%	8633 (68.5)	899 (66.2)	6172 (68.6)	1562 (69.1)	
Slightly, 40–49%	3050 (24.2)	340 (25.0)	2193 (24.4)	517 (22.9)	
Moderately, 30–39%	797 (6.3)	99 (7.3)	546 (6.1)	152 (6.7)	
Severely, <30%	132 (1.0)	21 (1.5)	83 (0.9)	28 (1.2)	
Anterior myocardial infarction	9091 (56.2)	1121 (58.6)	6431 (56.1)	1539 (55.0)	0.048
Three-vessel coronary artery disease^a^	3762 (36.4)	300 (29.2)	2624 (35.9)	838 (41.9)	<0.0001
**Laboratory variables**					
Creatinine, mmol/L	74.0 (61.7–90.0)	71.0 (59.0–87.0)	74.0 (62.0–90.0)	75.5 (62.0–91.0)	<0.0001
Glucose, mmol/L	7.0 (5.7–9.1)	6.4 (5.4–7.6)	6.8 (5.6–8.7)	9.2 (6.8–12.8)	<0.0001
Glycated hemoglobin A1c, %	6.0 (5.5–7.0)	5.6 (5.3–6.0)	5.9 (5.5–6.5)	6.9 (6.0–8.3)	<0.0001
Total cholesterol, mmol/L	4.5 (3.8–5.3)	4.1 (3.6–4.6)	4.5 (3.8–5.2)	5.3 (4.4–5.9)	<0.0001
Triglycerides, mmol/L	1.4 (1.0–2.0)	1.1 (0.8–1.6)	1.4 (1.0–2.0)	1.7 (1.2–2.6)	<0.0001
LDL cholesterol, mmol/L	2.8 (2.2–3.4)	2.4 (2.0–2.8)	2.7 (2.2–3.3)	3.4 (2.6–3.8)	<0.0001
HDL cholesterol, mmol/L	1.1 (0.9–1.3)	1.1 (0.9–1.3)	1.1 (0.9–1.3)	1.1 (0.9–1.3)	0.16

*SMuRF* Standard modifiable cardiovascular risk factor, *LVEF* Left ventricular ejection fraction

^a^Information on three-vessel coronary artery disease was only available for patients who conducted coronary angiography (*n* = 11045)

**Table 3:** Medical management and in-hospital outcomes of patients with first ST-segment elevation myocardial infarction by number of cardiovascular risk factors

Variables	Total (***N*** = 16228)	No SMuRF (***N*** = 1918)	1–2 SMuRFs (***N*** = 11503)	3–4 SMuRFs (***N*** = 2807)	***P*** value
**In-hospital management**					
Reperfusion therapy					
Among all the patients					<0.0001
No reperfusion	7430 (46.2)	1000 (52.8)	5323 (46.7)	1107 (39.8)	
Fibrinolysis	1618 (10.1)	172 (9.1)	1173 (10.3)	273 (9.8)	
Primary PCI	7017 (43.7)	722 (38.1)	4894 (43.0)	1401 (50.4)	
Among patients admitted within 12 h from onset					<0.0001
No reperfusion	2953 (27.6)	394 (33.2)	2118 (28.0)	441 (22.6)	
Fibrinolysis	1552 (14.5)	163 (13.7)	1122 (14.8)	267 (13.7)	
Primary PCI	6210 (58.0)	631 (53.1)	4336 (57.2)	1243 (63.7)	
Door-to-balloon time, minute	107 (75–159)	110 (63–200)	107 (75–158)	105 (75–150)	0.75
Door-to needle time, minute	53 (30–90)	50 (29–80)	55 (30–90)	50 (30–101)	0.30
Any PCI	10361 (63.8)	1041 (54.3)	7342 (63.8)	1978 (70.5)	<0.0001
Any CABG	98 (0.6)	9 (0.5)	65 (0.6)	24 (0.9)	0.15
Intra-aortic balloon pump use	547 (3.4)	50 (2.6)	374 (3.3)	123 (4.4)	0.002
**Medication during hospitalization**					
Aspirin	15697 (97.1)	1840 (96.6)	11119 (97.0)	2738 (97.8)	0.03
P_2_Y_12_-receptor inhibitor	15669 (97.5)	1811 (95.7)	11128 (97.7)	2730 (98.1)	<0.0001
Statin	15598 (96.8)	1807 (95.6)	11076 (96.9)	2715 (97.2)	0.006
β-blocker	11227 (70.0)	1270 (67.2)	7935 (69.7)	2022 (73.4)	<0.0001
ACEI/ARB	9404 (58.7)	914 (48.5)	6619 (58.2)	1871 (67.9)	<0.0001
Heparin	14449 (91.5)	1642 (88.8)	10288 (91.8)	2519 (92.2)	<0.0001
Glucoprotein IIb or IIIa inhibitor	5511 (35.3)	549 (30.0)	3920 (35.3)	1042 (38.5)	<0.0001
**In-hospital outcome**					
Death	1015 (6.3)	157 (8.2)	702 (6.1)	156 (5.6)	0.0006
Cardiac arrest	567 (3.5)	90 (4.8)	394 (3.5)	83 (3.0)	0.004
Heart failure	2501 (15.6)	311 (16.5)	1778 (15.6)	412 (14.8)	0.31
Re-infarction	88 (0.5)	8 (0.4)	62 (0.5)	18 (0.6)	0.59
Cerebrovascular accident or stroke	127 (0.8)	12 (0.6)	92 (0.8)	23 (0.8)	0.72
Severe arrhythmia	1372 (8.5)	161 (8.5)	1005 (8.8)	206 (7.4)	0.06
Length of stay, day	10 (7–13)	10 (7–14)	10 (7–13)	9 (7–13)	0.04
**Medication at discharge**^a^					
Aspirin	15066 (99.2)	1726 (98.3)	10706 (99.3)	2634 (99.4)	<0.0001
Clopidogrel	14868 (98.0)	1701 (97.1)	10567 (98.0)	2600 (98.2)	0.03
Statin	14993 (98.7)	1709 (97.5)	10664 (98.9)	2620 (98.9)	<0.0001
β-blocker	11725 (77.3)	1319 (75.2)	8279 (76.9)	2127 (80.5)	<0.0001
ACEI/ARB	10135 (66.8)	1014 (57.9)	7131 (66.2)	1990 (75.3)	<0.0001

* SMuRF* standard modifiable cardiovascular risk factor, *PCI* Percutaneous coronary intervention, *CABG* Coronary artery bypass graft, *ACEI* Angiotensin-converting enzyme inhibitor, *ARB* angiotensin receptor blocker

^a^Medication at discharge was described after removing patients with in-hospital deaths

**Fig. 1:**
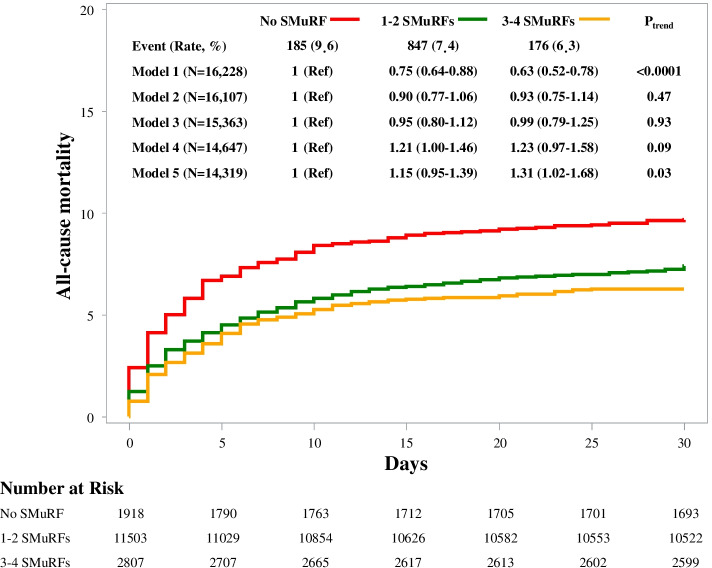
Survival curves for the cumulative incidence rate of all-cause mortality to 30 days. Model 1 was unadjusted; model 2 was adjusted for age only; model 3 was adjusted for age, sex, education, hospital levels, BMI, family history of CAD, prior history of stroke, prior history of COPD, and pre-admission aspirin; model 4 was further adjusting for presenting characteristics including onset-to-arrival time, pre-admission cardiac arrest, heart rate, systolic blood pressure, plasma creatinine, Killip class, and anterior myocardial infarction; model 5 was further adjusted for in-hospital medical treatment including reperfusion strategies and evidence-based medications (aspirin, P2Y12-receptor inhibitor, statin, ACEI/ARB, and β-blocker). SMuRF, standard modifiable cardiovascular risk factor; CAD, coronary artery disease; COPD, chronic obstructive pulmonary disease; ACEI, angiotensin-converting enzyme inhibitor; ARB, angiotensin receptor blocker

The mortality rate at 30 days after STEMI presentation was also significantly higher among patients without any SMuRF (9.6%, 7.4%, and 6.3% for 0, 1–2, and 3–4 SMuRF groups, respectively; Fig. 1). Compared with patients without any SMuRF, those with 1–2 SMuRFs and 3–4 SMuRFs were associated with an HR of 0.75 (95% CI, 0.64–0.88) and 0.63 (0.52–0.78) for all-cause mortality up to 30 days in the unadjusted model (*P* for trend < 0.0001). However, after adjusting for age, the increased risk hazard of 30-day mortality in patients without SMuRFs was eliminated (HR for 1–2 SMuRFs, 0.90 [0.77–1.06]; HR for 3–4 SMuRFs, 0.93 [0.75–1.14]; *P* for trend = 0.47), and the results were not materially changed with additional adjustment of other baseline characteristics and medical history. When further adjusting for presenting characteristics, we found that the association was reversed but the trend did not reach statistical significance. After further adjustment of in-hospital treatments, the association between numbers of SMuRFs and mortality risk was completely reversed. Compared with those with no SMuRFs, patients with more SMuRFs experienced a higher mortality risk (HR for 1–2 SMuRFs, 1.15 [0.95–1.39]; HR for 3–4 SMuRFs, 1.31 [1.02–1.68]; *P* for trend = 0.03). We also assessed the association of SMuRFs and in-hospital mortality and observed similar results with the primary outcome (Additional file 1: Table S3). For clinical outcomes up to 2 years, we observed that the unadjusted all-cause mortality persisted to be higher among patients without SMuRFs (Additional file 1: Fig. S4), while the Landmark analysis showed that the events that contributed to the difference in mortality rates at 2 years almost occurred within the first 30 days (Additional file 1: Fig. S4). For patients surviving up to 30 days, there was no significant difference in mortality rates throughout the 2-year follow-up period. After multivariate adjustment, patients with SMuRFs were more likely to experience a higher risk of all-cause mortality (Additional file 1: Table S4).

In stratified analysis, the association of the number of SMuRFs with mortality risk after multivariate adjustment was modified by time from symptom onset (Table 4). Among patients arriving in hospitals beyond 12 h from onset, patients with more SMuRFs experienced a significantly higher mortality risk (HR for 1–2 SMuRFs, 1.39 [1.03–1.87]; HR for 3–4 SMuRFs, 2.06 [1.41–3.01]), whereas the number of SMuRFs was not significantly associated with 30-day mortality among their counterparts (*P* for interaction = 0.01). Several sensitivity analyses were conducted to address the potential influence of risk factor misclassification caused by incomplete information or underdiagnosis. Considering that patients with more severe conditions might be unable to provide complete or reliable information on history risk factors, we also conducted a stratified analysis by severity of disease and found that the difference in crude mortality risk by the number of SMuRFs was significant among patients at low-risk levels (Table 4). When excluding patients who died within the first 24 h or those who transferred out, the difference in crude mortality risk across groups was attenuated, and the point estimates of HRs in adjusted models were materially unchanged but the confidence interval became statistically insignificant due to decreased event numbers (Additional file 1: Table S5). When excluding patients without complete information on lipid measurements and glucose concentration, we found that the difference in crude mortality between groups attenuated (HR for 1–2 SMuRFs, 0.81 [0.66–1.00]; HR for 3–4 SMuRFs, 0.80 [0.63–1.03]), but the association between SMuRFs status and mortality risk in adjusted models was strengthened (HR for 1–2 SMuRFs, 1.15 [0.92–1.44]; HR for 3–4 SMuRFs, 1.45 [1.10–1.91]).

**Table 4:** Subgroup analysis for all-cause mortality at 30 days

	Event/total (%)	Unadjusted models			Adjusted models		
		1–2 SMuRFs vs no SMuRFs	3–4 SMuRFs vs no SMuRFs	*P*_interaction_	1–2 SMuRFs vs no SMuRFs	3–4 SMuRFs vs no SMuRFs	*P*_interaction_
**Age**				0.27			0.20
<55	129/4834 (2.7)	0.85 (0.49–1.49)	0.67 (0.34–1.32)		1.76 (0.81–3.81)	1.79 (0.73–4.39)	
55–<75	597/8659 (6.9)	0.95 (0.74–1.22)	0.86 (0.63–1.16)		1.38 (1.03–1.87)	1.38 (0.96–1.99)	
≥75	466/2614 (17.8)	0.77 (0.62–0.97)	0.97 (0.70–1.35)		0.90 (0.69–1.18)	1.27 (0.85–1.89)	
**Sex**				0.07			0.88
Male	708/12381 (5.7)	0.76 (0.61–0.94)	0.59 (0.45–0.77)		1.13 (0.87–1.47)	1.25 (0.89–1.75)	
Female	500/3847 (13.0)	0.86 (0.68–1.09)	0.93 (0.68–1.27)		1.18 (0.90–1.56)	1.46 (1.01–2.11)	
**Hospital class**				0.65			0.96
Province-level	251/5256 (4.8)	1.01 (0.66–1.55)	0.87 (0.53–1.43)		1.13 (0.70–1.82)	1.19 (0.67–2.10)	
Prefecture-level	670/8808 (7.6)	0.76 (0.61–0.94)	0.64 (0.48–0.84)		1.13 (0.88–1.45)	1.32 (0.95–1.85)	
County-level	287/2164 (13.3)	0.73 (0.54–0.99)	0.75 (0.50–1.12)		1.38 (0.93–2.03)	1.69 (1.01–2.83)	
**Time from onset**				0.055			0.01
<12 h	694/10818 (6.4)	0.69 (0.56–0.85)	0.52 (0.40–0.69)		0.99 (0.77–1.27)	0.97 (0.70–1.35)	
≥12 h	503/5284 (9.5)	0.85 (0.66–1.09)	0.87 (0.63–1.20)		1.39 (1.03–1.87)	2.06 (1.41–3.01)	
**Primary PCI**				0.89			0.39
Yes	248/7017 (3.5)	0.77 (0.53–1.13)	0.75 (0.48–1.17)		0.85 (0.55–1.30)	0.95 (0.57–1.59)	
No	952/9069 (10.5)	0.78 (0.65–0.92)	0.69 (0.54–0.87)		1.24 (1.00–1.53)	1.41 (1.06–1.87)	
**Killip class**				0.07			0.10
I–II	866/14976 (5.8)	0.69 (0.58–0.83)	0.56 (0.44–0.72)		1.09 (0.88–1.34)	1.37 (1.02–1.83)	
III–IV	332/1181(28.1)	1.07 (0.77–1.50)	0.88 (0.59–1.32)		1.47 (0.95–2.28)	1.51 (0.91–2.52)	
**LVEF<40%**				0.29			0.49
No	362/11683 (3.1)	0.83 (0.61–1.14)	0.62 (0.41–0.92)		1.06 (0.76–1.49)	1.01 (0.64–1.60)	
Yes	126/929 (13.6)	1.08 (0.62–1.87)	1.11 (0.58–2.10)		1.32 (0.70–2.46)	1.23 (0.56–2.68)	
**GRACE score**				0.88			0.89
≤140	124/6933 (1.8)	0.83 (0.46–1.49)	0.81 (0.41–1.58)		0.95 (0.50–1.79)	1.13 (0.54–2.38)	
>140	949/8500 (11.2)	0.94 (0.78–1.14)	0.97 (0.77–1.23)		1.15 (0.94–1.40)	1.30 (0.99–1.69)	

Models were adjusted for age, sex, education, hospital levels, BMI, family history of CAD, prior history of stroke, prior history of COPD, pre-admission aspirin, onset-to-arrival time, pre-admission cardiac arrest, heart rate, systolic blood pressure, plasma creatinine, Killip class, anterior myocardial infarction, reperfusion strategies, and evidence-based medications (aspirin, P2Y12-receptor inhibitor, statin, ACEI/ARB, and β-blocker)

*SMuRF* Standard modifiable cardiovascular risk factor, *PCI* Percutaneous coronary intervention, *CAD* Coronary artery disease, *COPD* Chronic obstructive pulmonary disease, *ACEI* Angiotensin-converting enzyme inhibitor, *ARB* Angiotensin receptor blocker

## Discussion

In this nationwide registry, we found that the number of SMuRFs was inversely associated with an increased risk of 30-day all-cause mortality among patients with first-presentation STEMI. However, after accounting for marked differences in age, pre-admission characteristics, clinical profiles at presentation, and in-hospital management, the direction of the association was completely altered and the multivariate analysis suggested that the number of SMuRFs itself was associated with a higher risk of mortality. Our study suggested that the paradoxical association between the number of SMuRFs and crude mortality risk in clinical practice was not biological and could be possibly explained by bias from confounding factors.

Recently, the term SMuRF-less has been coined to raise awareness of this challenging group of patients without any SMuRF which was overlooked in clinical publications and guidelines but experienced higher mortality rates compared with those with at least one SMuRF [4, 7, 13]. Previous studies from highly developed countries documented that there was a large proportion of patients presenting STEMI without any SMuRFs (14.5% in Canada [8], 14.9% in Sweden [7], 19% in Australia [13], and 23.1% in Japan) [11], with an increasing trend observed in some countries [6, 13]. In this study, we observed that the proportion was a little bit lower in China (11.8%), with the estimate comparable to that in the China PEACE-Retrospective Acute Myocardial Infarction Study (9.7%) [21]. Moreover, the China PEACE study even reported a significant decrease in the proportion of patients with no SMuRFs at the time of admission from 2011 to 2015 (11 to 5.5%) [16, 21]. Nevertheless, considering the total number of STEMI patients almost nearly doubled in China at the same period [16], there was a substantial absolute number of patients presenting life-threatening STEMI without any SMuRF who cannot benefit from primary prevention strategies.

Similar to previous reports [7–10], there was also a striking inverse association between the number of SMuRFs and crude mortality rates among STEMI patients in our study, whereas the difference might reflect the more favorable profiles among patients with SMuRFs, and most importantly their younger age. Similar to other paradoxes (smoking and obesity) [22–25], patients with more SMuRFs might have faster progression of the CAD, while patients without any SMuRF presented with STEMI at a later age with an increased absolute baseline hazard. Thus, the substantial difference in age explained most of the observed higher 30-day mortality among those with no SMuRF, who were on average 5 years older than those with 3–4 risk factors. After adjusting for age, we found that patients with different numbers of SMuRFs were almost at a similar risk of dying. Besides, patients with fewer SMuRFs were also accompanied with other poor risk profiles (more females, poorly educated, and admitted to county-level hospitals). Previous studies have reported that those unfavorable factors, as well as the existence of those individual components of SMuRFs, were associated with longer pre-hospital delay [26–28], which was consistent with our finding that patients with no SMuRF experienced significantly longer time from onset to FMC compared with their counterparts. Thus, this might also explain the clear differences in severity of illness at admission that patients with fewer SMuRFs presented with higher GRACE risk scores. Furthermore, collaborating with prior studies [8, 10], our study also found large disparities in clinical management that patients with fewer SMuRFs were less likely to receive primary PCI and evidence-based medications during hospitalization. Ultimately, the unfavorable baseline risk factors, pre-hospital delays, presenting with high risk at admission, and suboptimal guideline-indicated treatments might collectively contribute to the increased crude mortality risk among patients with fewer SMuRFs. After adjusting for those aforementioned risk factors, we found that the direction of association was completely altered and the number of SMuRFs was positively associated with higher mortality risk.

However, some previous studies reported that the inverse association of the number of SMuRFs with mortality risk persisted even after multivariate adjustment, which might be related to inadequate control of residual confounding [10, 29]. Most recently, the SWEDEHEART study, a large prospective cohort enrolled 62,048 patients with first STEMI in Sweden, found that the association became neutral after adjusting for pharmacotherapy prescription at discharge (statin, ACEI/ARB, and β-blocker) [7]. This finding suggested that suboptimal prescription rates of evidence-based medications might be responsible for the increased 30-day mortality of no SMuRF. However, in the CAMI registry, we found that the association was completely altered after multivariate adjustment for demographics, presenting characteristics, and in-hospital management. A possible explanation could be the disparities in medical care systems. In the SWEDEHEART study, the median time from onset to intensive care was only 3.1 h and 71.4% of patients received primary PCI, whereas the median time from onset to first medical contact in the CAMI registry was 6.5 h, and the rate of primary PCI was also much lower (43.7%). Our subgroup analysis found that the adjusted association of the number of SMuRFs with mortality risk was only significant among patients admitted to hospitals beyond 12 h from the onset while being neutral among their counterparts. It is possible that patients timely admitted to hospitals could be more likely to receive effective reperfusion therapy. The analysis stratified by primary PCI also suggested that the adjusted association was not significant among patients receiving primary PCI, which was not surprising since several risk scores for predicting mortality after primary PCI for AMI patients also did not include these modifiable risk factors [30, 31]. Collectively, these findings implied that the early access to timely effective reperfusion might modify the poor prognosis caused by SMuRFs. This also explains why the number of SMuRFs was positively associated with mortality after multivariate adjustment in our study but not in previous reports from developed countries with high-quality medical systems [7, 9]. Our subgroup analysis stratified by hospital levels also supports this hypothesis that the adjusted association was less pronounced in province-level or prefecture-level hospitals compared with county-level hospitals although the interactive effect was not significant. Therefore, it is critical to improve the quality of the medical care system in these developing countries like China, especially for those remote areas with limited medical resources and low public awareness of ACS [15, 16, 32].

Our study evaluated the clinical characteristics, medical management, and outcomes among patients with different numbers of SMuRFs in a more diverse population where large differences in both risk perceptions in STEMI and clinical management exist. This study has several strengths including the nationally represented sample, standardized data collection, complete information on survival status, and extensive covariates for adjustment. Our study also has some limitations. First, the ascertainment of risk factors was through self-reported information, laboratory measurements, and medical record review at admission. Considering the possibility that patients with severe presenting characteristics at admission might be unable to provide complete and reliable information on risk factors [10], we removed participants who died within the first 24 h or transferred out and conducted a stratified analysis by the severity of disease (Killip class, Grace score, and LVEF). We found that among those with low-level risk at admission, the findings were not materially changed. Moreover, it is possible that the patients at first presentation of STEMI were unaware of or have not received a diagnosis of risk factors. To address the influence of underdiagnosis of risk factors, we limited the analysis among patients with complete laboratory measurements on lipids and glucose. This analysis showed that the unadjusted association between the number of SMuRFs and mortality was attenuated while the adjusted effect size was more pronounced. Thus, the higher crude mortality risk among those with fewer SMuRFs might be somewhat influenced by the artifact from the increased risk caused by undiagnosed risk factors [33]. However, information on persisted hypertension during hospitalization and new diagnosis of risk factors at discharge were not available for the current study. Thus, the artifact from risk factor misclassification might be another reason for the increased crude mortality risk among patients without any SMuRF, which further supports our main conclusion that the fewer SMuRFs were not biologically associated with increased mortality risk. Besides, although the CAMI registry enrolled patients across all 31 provinces and municipalities in China with broad coverage of geographical regions, our study cannot be fully representative of all patients in China. However, the CAMI registry uniquely included 108 hospitals across three levels throughout mainland China, which might comprehensively represent the vertical administrative models in the medical system and objectively portray the characteristics of the routine practice of medical care for STEMI patients in China. Moreover, our study only included patients admitted to hospitals, and there was no information on those who did not admit to hospitals (outpatients or patients who died before admitting to hospitals), which might have led to potential selection bias. Finally, although we have adjusted for a comprehensive set of covariates, the possibility of unmeasured confounders cannot be excluded in such an observational study.

## Conclusions

In summary, our study indicates that patients with fewer SMuRFs admitted with first-presentation STEMI had more unfavorable risk profiles and were less aggressively managed with evidence-based therapies. The increased mortality risk among patients without SMuRFs is explained by confounding factors related to their poor risk profiles (older age, longer pre-hospital delays, and poorer clinical management). After adjusting for confounding factors, a higher risk-factor burden translates into poor prognosis among patients with STEMI but the association can be modified by timely medical care. Our study strongly reinforces the influence of traditional risk factors on the individual prognosis in clinical practice and the importance of achieving the earliest access to timely effective medical care.

## Supplementary Information

**Additional file 1: Figure S1.** Chinese vertical governmental and administrative model and the three-level hospitals in the CAMI registry. **Figure S2.** Flow-chart of patients included in this study. **Figure S3.** Reasons for No Reperfusion Therapy among the Eligible STEMI Patients. **Figure S4.** Landmark analysis of association of SMuRF status with all-cause mortality to 2 years. **Table S1.** Definition of study variables. **Table S2.** Baseline information stratified by the number of standard modifiable risk factors. **Table S3.** Adjusted Proportions of Evidence-based Medication Use among Patients with First ST-segment Elevation Myocardial Infarction by Number of Cardiovascular Risk Factors. **Table S4.** Association of SMuRF status with in-hospital, 30-day, and 2-year mortality. **Table S5.** Sensitivity analysis for the association of SMuRF status with all-cause mortality at 30 days.

## Data Availability

Data described in the manuscript and the analytical code will be made available during the CAMI study conduct only to the investigators who have participated in/contributed to the study.

## Abbreviations

ACEIs: Angiotensin-converting enzyme inhibitors
AMI: Acute myocardial infarction
ARB: Angiotensin receptor blockers
CABG: Coronary artery bypass graft
CAD: Coronary artery disease
CAMI: China Acute Myocardial Infarction registry
PCI: Percutaneous coronary intervention
SMuRFs: Standard modifiable risk factors
STEMI: ST-elevation myocardial infarction
