# Supplementary material for: Number of standard modifiable risk factors and mortality in patients with first-presentation ST-segment elevation myocardial infarction: insights from China Acute Myocardial Infarction registry
Source: BMC Med. 2022 Jul 6;20:217. doi: 10.1186/s12916-022-02418-w (PMC9258075; doi:10.1186/s12916-022-02418-w)
Supplement: Supplementary file 1 — Additional file 1: Figure S1. Chinese vertical governmental and administrative model and the three-level hospitals in the CAMI registry. Figure S2. Flow-chart of patients included in this study. Figure S3. Reasons for No Reperfusion Therapy among the Eligible STEMI Patients. Figure S4. Landmark analysis of association of SMuRF status with all-cause mortality to 2 years. Table S1. Definition of study variables. Table S2. Baseline information stratified by the number of standard modifiable risk factors. Table S3. Adjusted Proportions of Evidence-based Medication Use among Patients with First ST-segment Elevation Myocardial Infarction by Number of Cardiovascular Risk Factors. Table S4. Association of SMuRF status with in-hospital, 30-day, and 2-year mortality. Table S5. Sensitivity analysis for the association of SMuRF status with all-cause mortality at 30 days. [file 12916_2022_2418_MOESM1_ESM.docx]

**Additional File 1: Supplementary tables and figures**

**Figure S1. Chinese vertical governmental and administrative model and the three-level hospitals in the CAMI registry.**


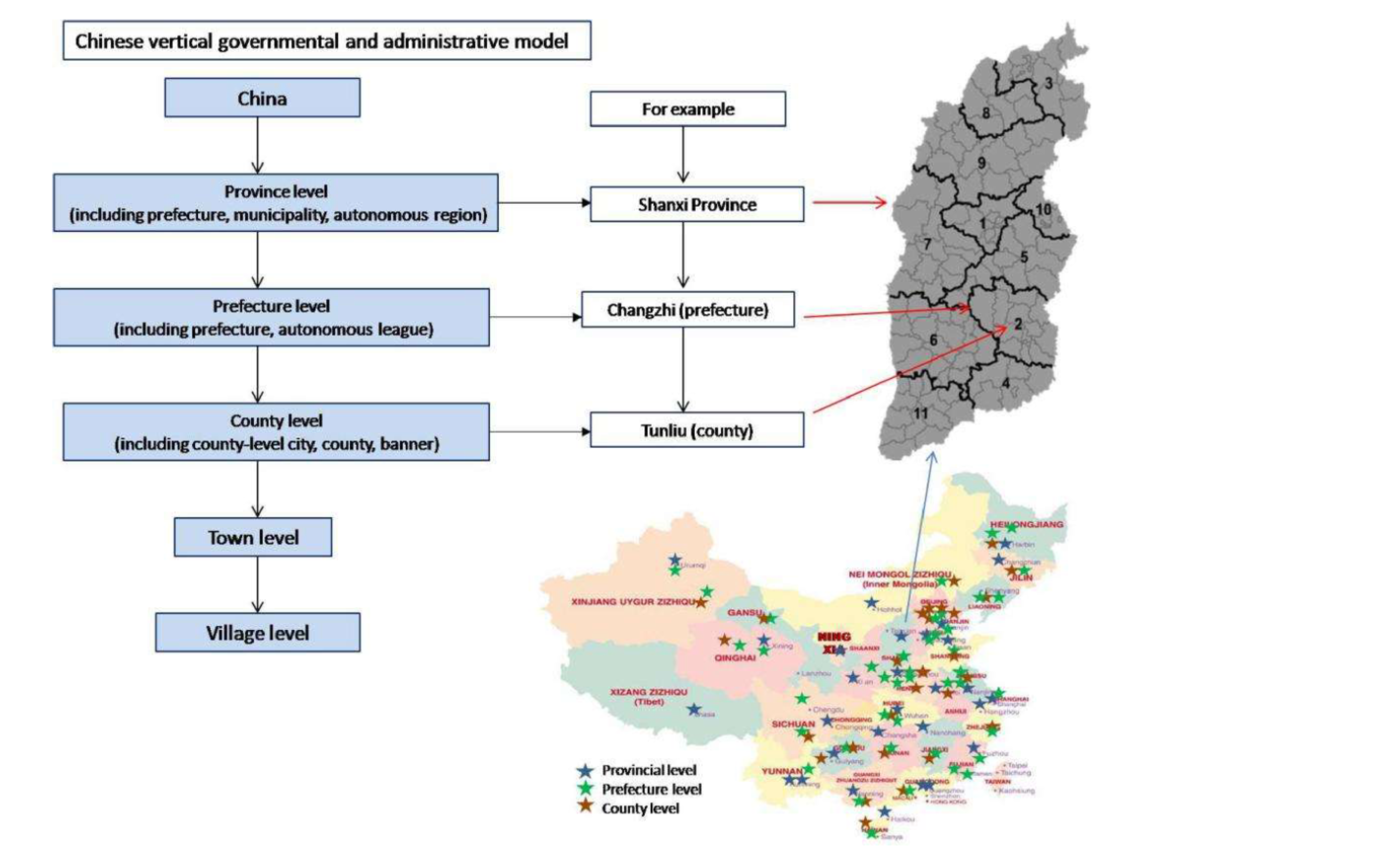


**Figure S2. Flow-chart of patients included in this study.** STEMI, ST-elevation myocardial infarction; CAD, coronary artery disease; MI, myocardial infarction; PCI, percutaneous coronary intervention; CABG, coronary artery bypass graft.


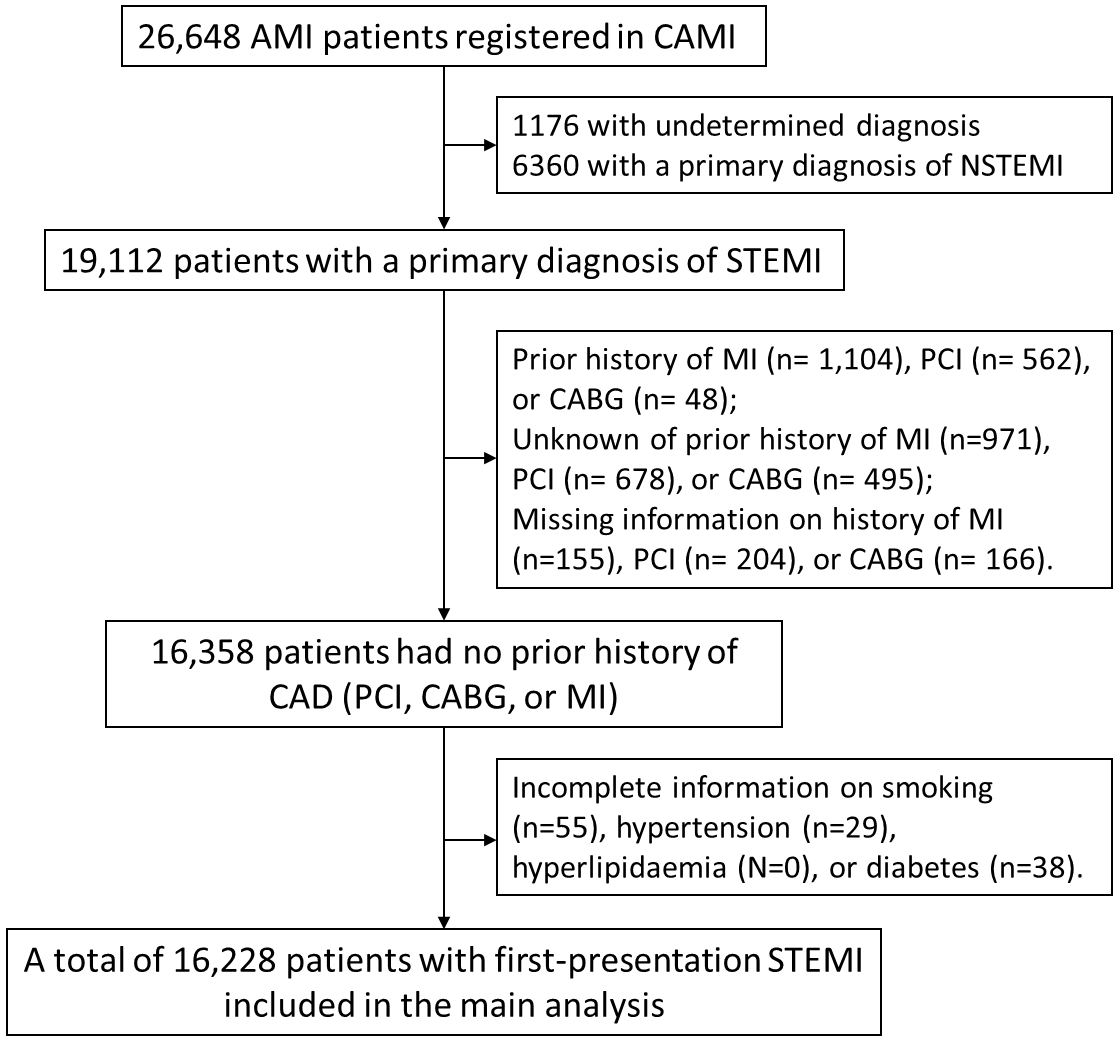


**Figure S3. Reasons for No Reperfusion Therapy among the Eligible STEMI Patients**

**Figure S4. Landmark analysis of association of SMuRF status with all-cause mortality to 2 years.** The top and bottom panel shows cumulative incident rate of all-cause mortality to 2 years among the overall patients and patients surviving at 30 days, respectively. SMuRF=standard modifiable cardiovascular risk factor.


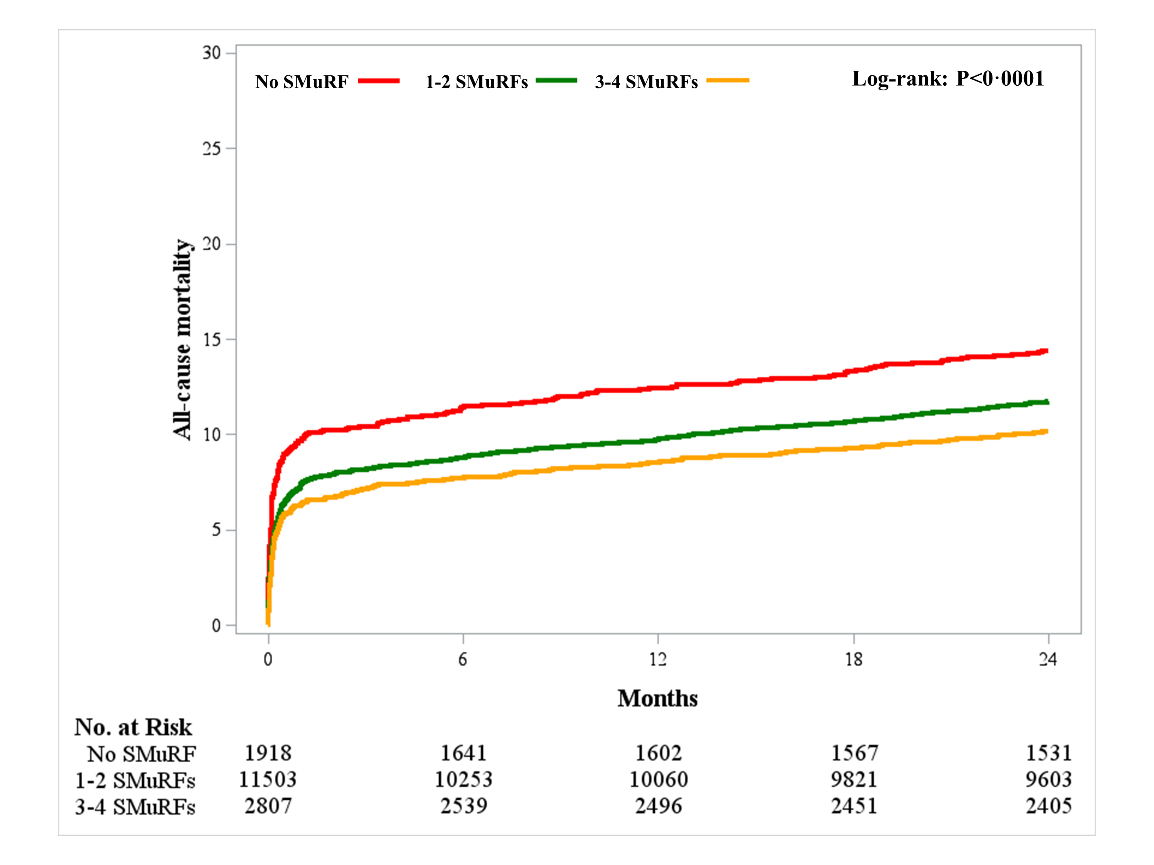


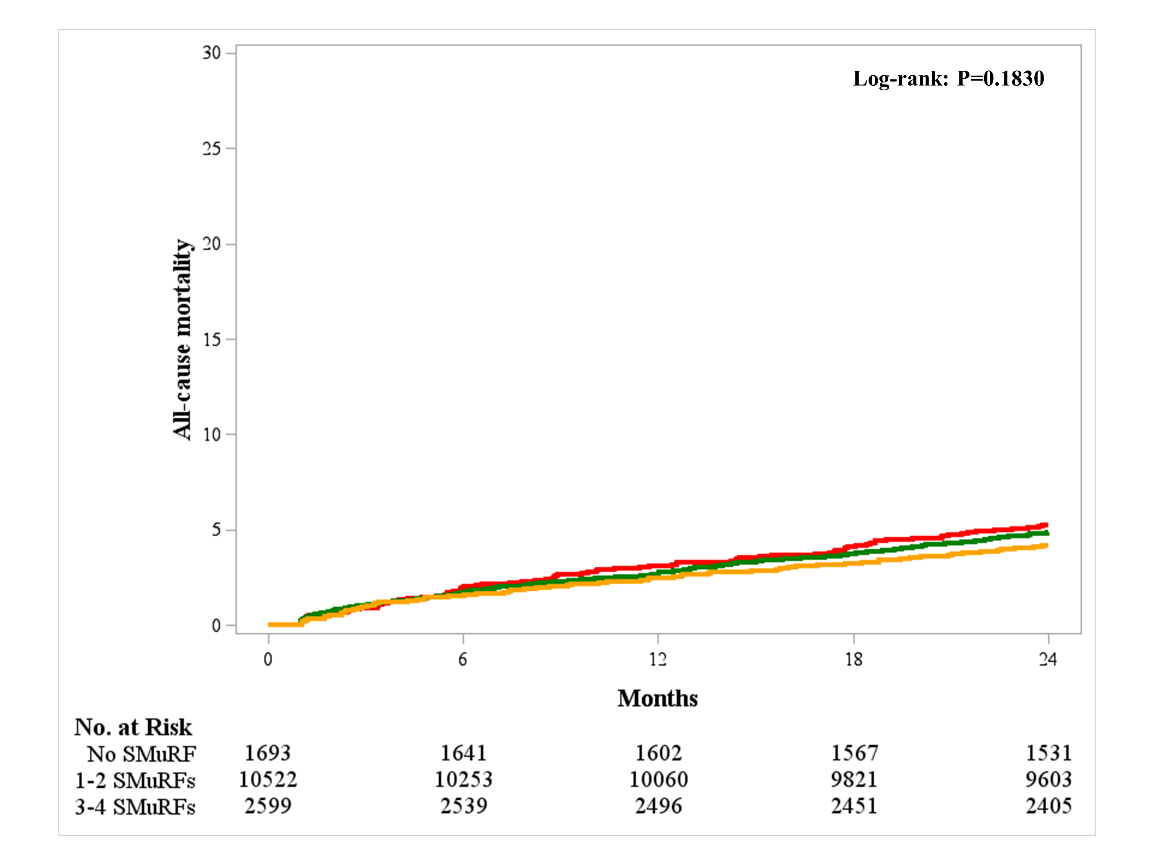


**Table S1. Definition of study variables**

| **Variables** | **Definition** | **Continuous/**  **Categorical/ Binary** | **Completeness (%)** |
| --- | --- | --- | --- |
| **Admission characteristics** |  |  |  |
| Age* | Years after birth | Continuous | 99.3 |
| Sex* | Female/Male | Binary | 100 |
| Education* | Education level; Illiterate/Primary or secondary/High/unknown | Categorical | 100 |
| Hospital class* | Province-level/Prefecture-level/County-level | Categorical | 100 |
| Smoking status | Current smoking was defined as smoking regularly within the past month before admission; current/former/never | Binary | 100 |
| Hypertension | Self-reported hypertension or using antihypertensive medications before admission | Binary | 100 |
| Diabetes | Self-reported diabetes, a glucose concentration of 11.1 mmol/L or higher, or a glycated haemoglobinA1c of 6.5% or higher. | Binary | 100 |
| Hyperlipidemia | Self-reported hyperlipidaemia, using lipid-lowering medications before admission, an LDL-C concentration of 3.37 mmol/L or higher, or a total cholesterol concentration of 5.18 mmol/L or higher during hospitalization | Binary | 100 |
| Body-mass index, kg/m^2^ | Body mass index calculated during hospitalization, kg/m^2^ | Continuous | 96.3 |
| **Medical history** |  |  |  |
| Family history of coronary artery disease* | Family members (parents, siblings, and children) has ever been diagnosed with angina, acute myocardial infarction, sudden cardiac death without obvious cause, coronary artery bypass grafting, or percutaneous coronary intervention before 55 years for male and 50 years for female. Yes; No; Unknown | Categorical | 100 |
| Heart failure | Previous history of heart failure | Binary | 99.9 |
| Stroke* | Previous history of stroke | Binary | 99.9 |
| Peripheral arterial disease | Previous history of peripheral vascular disease (includes upper and lower extremity, renal, mesenteric, and abdominal aortic systems) | Binary | 99.9 |
| Chronic renal failure | Previous history of chronic renal failure | Binary | 99.7 |
| Chronic obstructive pulmonary disease* | Previous history of chronic obstructive pulmonary disease | Binary | 99.4 |
| **Pre-hospital pharmacotherapy** |  |  |  |
| Aspirin | Aspirin use in the past week before symptom onset | Binary | 99.4 |
| P_2_Y_12_ inhibitor | Use of any kinds of P2Y_12_ inhibitors in the past week before symptom onset | Binary | 99.2 |
| Statin | Use of any kinds of statins in the past week before symptom onset | Binary | 97.9 |
| β-blocker | Use of any kinds of β-blockers in the past week before symptom onset | Binary | 98.7 |
| ACEI/ARB | Use of any kinds of ACEIs/ARBs in the past week before symptom onset | Binary | 98.5 |
| **Presentation characteristics** |  |  |  |
| Means of transport | Ambulance; self or family; transferring in; in hospital. | Categorical | 99.7 |
| Onset-to-arrival time* | Time from symptom onset to arriving hospital | Categorical; <3, 3-12, 12-24, >24h | 99.2 |
| Time for symptoms to first medical contact, minute | Time from symptom onset to first contact with medical system (EMS system or arriving at hospitals) | Continuous | 80 |
| GRACE risk score | Evaluated on admission |  | 95.1 |
| Systolic blood pressure, mmHg | SBP measured on admission | Continuous | 99.2 |
| Heart rate, beats/min | Heart rate measured on admission | Continuous | 99.4 |
| Cardiac arrest at presentation | Cardiac arrest before admitting to hospitals or during the process of transport. |  | 100 |
| Killip class | Evaluated on admission | Categorical | 99.6 |
| LVEF | The first measurement of LVEF after hospitalization | Continuous | 77.7 |
| Anterior myocardial infarction | Evaluated on admission | Categorical | 99.7 |
| Three-vessel coronary artery disease | Evaluated using coronary angiography on admission. | Categorical | 93.7 |
| **Laboratory variables** |  |  |  |
| Creatinine, mmol/L | The first measurement of creation after hospitalization | Continuous | 96.7 |
| Glucose, mmol/L | The first measurement of glucose after hospitalization | Continuous | 96.1 |
| Glycated haemoglobin A1c, % | The first measurement of HbA1C after hospitalization | Continuous | 34.6 |
| Total cholesterol, mmol/L | Admission TC measured within 24 hours of hospitalization | Continuous | 90.6 |
| Triglycerides, mmol/L | Admission TG measured within 24 hours of hospitalization | Continuous | 91.6 |
| LDL cholesterol, mmol/L | Admission LDL-C measured within 24 hours of hospitalization | Continuous | 89.6 |
| HDL cholesterol, mmol/L | Admission HDL-C measured within 24 hours of hospitalization | Continuous | 89.6 |
| **In-hospital management** |  |  |  |
| Reperfusion therapy* | Reperfusion strategy on admission, No reperfusion/Fibrinolysis/Primary PCI | Categorical | 99 |
| Door-to-balloon time, minute | Time between the arrival in the emergency room until the time that a balloon is inflated in the occluded, culprit coronary artery | Continuous | 29.5 |
| Door-to needle time, minute | Time between the arrival in the emergency room until the time that the (bolus of) rtPA was given | Continuous | 53.6 |
| Any PCI | Patients receiving any PCI during hospitalization | Binary | 100 |
| Any CABG | Patients receiving any CABG during hospitalization | Binary | 100 |
| Intra-aortic balloon pump use | Use of IABP during hospitalization | Binary | 98.7 |
| **Medication during hospitalization** |  |  |  |
| Aspirin* | Use of aspirin during hospitalization | Binary | 99.6 |
| P_2_Y_12_-receptor inhibitor* | Use of any kinds of P2Y_12_ inhibitors in the past 2 weeks before symptom onset | Binary | 99 |
| Statin* | Use of any kinds of statins during hospitalization | Binary | 99.3 |
| β-blocker* | Use of any kinds of β-blocker during hospitalization | Binary | 98.8 |
| ACEI/ARB* | Use of any kinds of ACEI/ARB during hospitalization | Binary | 98.7 |
| Heparin | Use of heparin during hospitalization (not including during the process of PCI) | Binary | 97.3 |
| Glucoprotein IIb or IIIa inhibitor | Use of glucoprotein IIb or IIIa inhibitor during hospitalization | Binary | 96.3 |
| **In-hospital outcome** |  |  |  |
| Death | Any death during hospitalization | Binary | 100 |
| Cardiac arrest | Indicate if the patient experienced an episode of cardiac arrest in the facility. | Binary | 98.9 |
| Heart failure | Indicate if there is physician documentation or report of either new onset or acute reoccurrence of heart failure. | Binary | 99 |
| Re-infarction | Indicate if there are clinical signs and symptoms of a new infarction or repeat infarction. | Binary | 98.9 |
| Cerebrovascular accident or stroke | Defined as loss of neurological function caused by an ischemic or hemorrhagic  event with residual symptoms at least 24 hours after onset or leading to death. | Binary | 98.9 |
| Severe arrhythmia | Indicate if the patient has a new episode or acute recurrence of arrhythmia in your facility documented by 1 of the following: Arial fibrillation/flutter; Supraventricular tachycardia requiring treatment (supraventricular tachycardia that requires cardioversion, drug therapy, or is sustained for greater than 1 minute); Ventricular tachycardia or ventricular fibrillation; Sinus pause or bradycardia; second-degree, or high-degree, or third-degree atrioventricular (AV) block | Binary | 99 |
| Length of stay, day | Time from admission to discharge | Continuous | 96.4 |
| **Medication at discharge** |  |  |  |
| Aspirin | Prescription of aspirin at discharge | Binary | 99.9 |
| Clopidogrel | Prescription of clopidogrel at discharge | Binary | 99.8 |
| Statin | Prescription of any kinds of statin at discharge | Binary | 99.8 |
| β-blocker | Prescription of any kinds of β-blocker at discharge | Binary | 99.7 |
| ACEI/ARB | Prescription of any kinds of ACEIs/ARBs at discharge | Binary | 99.7 |

*Included as covariates in the multivariate analysis.

**Table S2. Baseline information stratified by the number of standard modifiable risk factors**

| **Variables** | **Total (N=16228)** | **0**  **(N=1918)** | **1**  **(N=5852)** | **2**  **(N=5651)** | **3**  **(N=2411)** | **4**  **(N=396)** | **P Value** |
| --- | --- | --- | --- | --- | --- | --- | --- |
| **Admission characteristics** |  |  |  |  |  |  |  |
| Age | 61.7±12.5 | 64.9±13.0 | 62.3±12.9 | 61.3±12.2 | 59.6±11.6 | 57.4±10.2 | <0.0001 |
| <55 | 4834 (30.0) | 436 (22.9) | 1697 (29.2) | 1690 (30.2) | 850 (35.5) | 161 (41.1) |  |
| 55-74 | 8659 (53.8) | 1004 (52.6) | 3043 (52.4) | 3108 (55.5) | 1296 (54.1) | 208 (53.1) |  |
| ≥75 | 2614 (16.2) | 467 (24.5) | 1069 (18.4) | 804 (14.4) | 251 (10.5) | 23 (5.9) |  |
| Male | 12381 (76.3) | 1318 (68.7) | 4492 (76.8) | 4307 (76.2) | 1901 (78.8) | 363 (91.7) | <0.0001 |
| Education |  |  |  |  |  |  | <0.0001 |
| Illiterate | 1293 (8.0) | 232 (12.1) | 544 (9.3) | 409 (7.2) | 100 (4.1) | 8 (2.0) |  |
| Primary or secondary | 9073 (55.9) | 1052 (54.8) | 3350 (57.2) | 3121 (55.2) | 1347 (55.9) | 203 (51.3) |  |
| High | 1348 (8.3) | 112 (5.8) | 436 (7.5) | 519 (9.2) | 229 (9.5) | 52 (13.1) |  |
| Unknown | 4514 (27.8) | 522 (27.2) | 1522 (26.0) | 1602 (28.3) | 735 (30.5) | 133 (33.6) |  |
| Hospital class |  |  |  |  |  |  | <0.0001 |
| Province-level | 5256 (32.4) | 496 (25.9) | 1808 (30.9) | 1891 (33.5) | 889 (36.9) | 172 (43.4) |  |
| Prefecture-level | 8808 (54.3) | 1098 (57.2) | 3215 (54.9) | 3060 (54.1) | 1245 (51.6) | 190 (48.0) |  |
| County-level | 2164 (13.3) | 324 (16.9) | 829 (14.2) | 700 (12.4) | 277 (11.5) | 34 (8.6) |  |
| Smoking status |  |  |  |  |  |  | <0.0001 |
| Never | 7062 (43.5) | 1582 (82.5) | 2824 (48.3) | 2061 (36.5) | 595 (24.7) | 0 (0) |  |
| Former | 1454 (9.0) | 336 (17.5) | 618 (10.6) | 402 (7.1) | 98 (4.1) | 0 (0) |  |
| Current | 7712 (47.5) | 0 (0) | 2410 (41.2) | 3188 (56.4) | 1718 (71.3) | 396 (100) |  |
| Hypertension | 7887 (48.6) | 0 (0) | 1822 (31.1) | 3604 (63.8) | 2065 (85.7) | 396 (100) | <0.0001 |
| Diabetes | 4312 (26.6) | 0 (0) | 624 (10.7) | 1768 (31.3) | 1524 (63.2) | 396 (100) | <0.0001 |
| Hyperlipidemia | 6060 (37.4) | 0 (0) | 996 (17.0) | 2742 (48.5) | 1926 (79.88) | 396 (100) | <0.0001 |
| Weight, kg | 68.1±10.9 | 64.4±10.5 | 67.0±10.7 | 68.8±10.6 | 70.8±11.3 | 74.1±10.3 | <0.0001 |
| Body-mass index, kg/m^2^ | 24.1±3.1 | 23.2±3.0 | 23.8±3.1 | 24.3±3.0 | 24.9±3.3 | 25.5±3.2 | <0.0001 |
| **Medical history** |  |  |  |  |  |  |  |
| Family history of coronary artery disease | 562 (3.5) | 34 (1.8) | 157 (2.7) | 213 (3.8) | 131 (5.4) | 27 (6.8) | <0.0001 |
| Heart failure | 114 (0.7) | 11 (0.6) | 39 (0.7) | 43 (0.8) | 20 (0.8) | 1 (0.3) | 0.63 |
| Stroke | 1399 (8.6) | 95 (5.0) | 457 (7.8) | 565 (10.0) | 230 (9.6) | 52 (13.1) | <0.0001 |
| Peripheral arterial disease | 61 (0.4) | 5 (0.3) | 12 (0.2) | 29 (0.5) | 11 (0.5) | 4 (1.0) | 0.01 |
| Chronic renal failure | 112 (0.7) | 8 (0.4) | 28 (0.5) | 55 (1.0) | 20 (0.8) | 1 (0.3) | 0.006 |
| Chronic obstructive pulmonary disease | 271 (1.7) | 45 (2.4) | 105 (1.8) | 82 (1.5) | 32 (1.3) | 7 (1.8) | 0.055 |
| **Pre-hospital pharmacotherapy** |  |  |  |  |  |  |  |
| Aspirin | 937 (5.8) | 38 (2.0) | 223 (3.8) | 371 (6.6) | 251 (10.4) | 54 (13.7) | <0.0001 |
| P_2_Y_12_ inhibitor | 318 (2.0) | 15 (0.8) | 68 (1.2) | 127 (2.3) | 96 (4.0) | 12 (3.0) | <0.0001 |
| Statin | 942 (5.9) | 0 (0) | 105 (1.8) | 417 (7.5) | 355 (14.9) | 65 (16.8) | <0.0001 |
| β-blocker | 518 (3.2) | 0 (0) | 71 (1.2) | 232 (4.2) | 179 (7.5) | 36 (9.3) | <0.0001 |
| ACEI/ARB | 763 (4.8) | 0 (0) | 139 (2.4) | 331 (5.9) | 238 (10.0) | 55 (14.2) | <0.0001 |
| **Presentation characteristics** |  |  |  |  |  |  |  |
| Emergency medical system | 1736 (10.7) | 186 (9.7) | 597 (10.2) | 607 (10.8) | 297 (12.4) | 49 (12.5) | 0.003 |
| Onset-to-arrival time |  |  |  |  |  |  | <0.0001 |
| <3h | 3844 (23.9) | 392 (20.7) | 1298 (22.4) | 1383 (24.6) | 651 (27.2) | 120 (30.5) |  |
| 3-12h | 6974 (43.3) | 811 (42.9) | 2522 (43.4) | 2443 (43.5) | 1021 (42.6) | 177 (44.9) |  |
| 12-24h | 1665 (10.3) | 197 (10.4) | 619 (10.7) | 571 (10.2) | 238 (9.9) | 40 (10.2) |  |
| 1-7d | 3619 (22.5) | 491 (26.0) | 1368 (23.6) | 1216 (21.7) | 487 (20.3) | 57 (14.5) |  |
| Time for symptoms to first medical contact, minute | 328 (139-1124) | 370 (165-1441) | 330 (141-1200) | 310 (137-1060) | 300 (120-1020) | 265 (120-827) | 0.0001 |
| GRACE risk score | 148.4±35.4 | 156.1±35.7 | 150.2±35.0 | 146.7±34.7 | 143.8±37.1 | 138.9±32.6 | <0.0001 |
| Systolic blood pressure, mmHg | 127.3±24.9 | 120.5±22.1 | 125.3±23.9 | 129.0±25.1 | 132.9±26.7 | 134.0±28.7 | <0.0001 |
| Heart rate, beats/min | 77.4±18.1 | 76.8±18.0 | 76.9±18.1 | 77.4±17.9 | 78.8±18.6 | 79.6±18.3 | <0.0001 |
| Cardiac arrest at presentation | 207 (1.3) | 24 (1.3) | 69 (1.2) | 75 (1.3) | 38 (1.6) | 1 (0.3) | 0.23 |
| Killip class |  |  |  |  |  |  | 0.51 |
| I | 12466 (77.2) | 1484 (77.7) | 4498 (77.2) | 4348 (77.2) | 1834 (76.4) | 302 (76.5) |  |
| II | 2510 (15.5) | 276 (14.5) | 899 (15.4) | 906 (16.1) | 369 (15.4) | 60 (15.2) |  |
| III | 566 (3.5) | 68 (3.6) | 200 (3.4) | 185 (3.3) | 97 (4.0) | 16 (4.1) |  |
| IV | 615 (3.8) | 81 (4.2) | 226 (3.9) | 190 (3.4) | 101 (4.2) | 17 (4.3) |  |
| LVEF |  |  |  |  |  |  | 0.058 |
| Normal,>50% | 8633 (68.5) | 899 (66.2) | 3093 (68.5) | 3079 (68.8) | 1333 (69.6) | 229 (66.6) |  |
| Slightly,40-49% | 3050 (24.2) | 340 (25.0) | 1109 (24.6) | 1084 (24.2) | 433 (22.6) | 84 (24.4) |  |
| Moderately,30-39% | 797 (6.3) | 99 (7.3) | 276 (6.1) | 270 (6.0) | 123 (6.4) | 29 (8.4) |  |
| Severely,<30% | 132 (1.0) | 21 (1.5) | 38 (0.8) | 45 (1.0) | 26 (1.4) | 2 (0.6) |  |
| Anterior myocardial infarction | 9091 (56.2) | 1121 (58.6) | 3303 (56.6) | 3128 (55.5) | 1309 (54.5) | 230 (58.1) | 0.048 |
| Three-vessel coronary artery disease * | 3762 (36.4) | 300 (29.2) | 1159 (32.4) | 1465 (39.1) | 703 (41.2) | 135 (45.5) | <0.0001 |
| **Laboratory variables** |  |  |  |  |  |  |  |
| Creatinine, mmol/L | 74.0 (61.7-90.0) | 71.0 (59.0-87.0) | 73.6 (61.5-89.2) | 74.6 (62.0-90.0) | 75.6 (62.0-91.1) | 74.5 (62.6-89.0) | <0.0001 |
| Glucose, mmol/L | 7.0 (5.7-9.1) | 6.4 (5.4-7.6) | 6.6 (5.5-8.0) | 7.2 (5.8-9.7) | 8.8 (6.6-12.4) | 11.3 (8.5-14.1) | <0.0001 |
| Glycated haemoglobin A1c, % | 6.0 (5.5-7.0) | 5.6 (5.3-6.0) | 5.7 (5.4-6.1) | 6.0 (5.5-7.2) | 6.8 (5.9-8.2) | 7.4 (6.7-8.5) | <0.0001 |
| Total cholesterol, mmol/L | 4.5 (3.8-5.3) | 4.1 (3.6-4.6) | 4.3 (3.7-4.8) | 4.7 (4.0-5.5) | 5.2 (4.4-5.8) | 5.4 (4.9-6.1) | <0.0001 |
| Triglycerides, mmol/L | 1.4 (1.0-2.0) | 1.1 (0.8-1.6) | 1.3 (0.9-1.8) | 1.5 (1.0-2.2) | 1.7 (1.2-2.5) | 2.0 (1.4-3.0) | <0.0001 |
| LDL cholesterol, mmol/L | 2.8 (2.2-3.4) | 2.4 (2.0-2.8) | 2.6 (2.1-3.1) | 2.9 (2.3-3.6) | 3.3 (2.6-3.9) | 3.5 (2.8-3.8) | <0.0001 |
| HDL cholesterol, mmol/L | 1.1 (0.9-1.3) | 1.1 (0.9-1.3) | 1.1 (0.9-1.3) | 1.1 (0.9-1.3) | 1.1 (0.9-1.3) | 1.0 (0.9-1.2) |  |
| **In-hospital management** |  |  |  |  |  |  |  |
| Reperfusion therapy |  |  |  |  |  |  |  |
| Among all the patients |  |  |  |  |  |  | <0.0001 |
| No reperfusion | 7430 (46.2) | 1000 (52.8) | 2818 (48.6) | 2505 (44.8) | 955 (40.0) | 152 (38.5) |  |
| Fibrinolysis | 1618 (10.1) | 172 (9.1) | 584 (10.1) | 589 (10.5) | 236 (9.9) | 37 (9.4) |  |
| Primary PCI | 7017 (43.7) | 722 (38.1) | 2399 (41.4) | 2495 (44.6) | 1195 (50.1) | 206 (52.2) |  |
| Among patients admitted within 12 hours from onset |  |  |  |  |  |  | <0.0001 |
| No reperfusion | 2953 (27.6) | 394 (33.2) | 1103 (29.1) | 1015 (26.8) | 365 (22.1) | 76 (25.7) |  |
| Fibrinolysis | 1552 (14.5) | 163 (13.7) | 560 (1.8) | 562 (14.8) | 231 (14.0) | 36 (12.2) |  |
| Primary PCI | 6210 (58.0) | 631 (53.1) | 2125 (56.1) | 2211 (58.4) | 1059 (64.0) | 184 (62.2) |  |
| Door-to-balloon time, minute | 107 (75-159) | 110 (63-200) | 105 (70-150) | 110 (80-163) | 105 (75-150) | 110 (90-152) | 0.29 |
| Door-to needle time, minute | 53 (30-90) | 50 (29-79.5) | 50 (30-90) | 56.5 (30-90) | 52.5 (30-103) | 45 (29-68) | 0.49 |
| Any PCI | 10361 (63.8) | 1041 (54.3) | 3602 (61.6) | 3740 (66.2) | 1686 (69.9) | 292 (73.7) | <0.0001 |
| Any CABG | 98 (0.6) | 9 (0.5) | 29 (0.5) | 36 (0.6) | 19 (0.8) | 5 (1.3) | 0.20 |
| Intra-aortic balloon pump use | 547 (3.4) | 50 (2.6) | 186 (3.2) | 188 (3.4) | 106 (4.4) | 17 (4.4) | 0.01 |
| **Medication during hospitalization** |  |  |  |  |  |  |  |
| Aspirin | 15697 (97.1) | 1840 (96.6) | 5641 (96.8) | 5478 (97.3) | 2349 (97.8) | 389 (98.2) | 0.04 |
| P_2_Y_12_-receptor inhibitor | 15669 (97.5) | 1811 (95.7) | 5662 (97.7) | 5466 (97.6) | 2340 (98.0) | 390 (99.0) | <0.0001 |
| Statin | 15598 (96.8) | 1807 (95.6) | 5620 (96.8) | 5456 (97.0) | 2327 (97.0) | 388 (98.0) | 0.02 |
| β-blocker | 11227 (70.0) | 1270 (67.2) | 4033 (69.5) | 3902 (69.8) | 1718 (72.7) | 304 (77.9) | <0.0001 |
| ACEI/ARB | 9404 (58.7) | 914 (48.5) | 3240 (55.9) | 3379 (60.6) | 1593 (67.4) | 278 (71.1) | <0.0001 |
| Heparin | 14449 (91.5) | 1642 (88.8) | 5228 (91.6) | 5060 (91.9) | 2161 (92.0) | 358 (93.5) | 0.0003 |
| Glucoprotein IIb or IIIa inhibitor | 5511 (35.3) | 549 (30.0) | 1920 (34.1) | 2000 (36.6) | 892 (38.3) | 150 (39.5) | <0.0001 |
| **In-hospital outcome** |  |  |  |  |  |  |  |
| Death | 1015 (6.3) | 157 (8.2) | 360 (6.2) | 342 (6.1) | 147 (6.1) | 9 (2.3) | 0.0001 |
| Cardiac arrest | 567 (3.5) | 90 (4.8) | 202 (3.5) | 192 (3.4) | 77 (3.2) | 6 (1.5) | 0.008 |
| Heart failure | 2501 (15.6) | 311 (16.5) | 884 (15.3) | 894 (16.0) | 371 (15.5) | 41 (10.5) | 0.04 |
| Re-infarction | 88 (0.5) | 8 (0.4) | 30 (0.5) | 32 (0.6) | 18 (0.8) | 0 (0) | 0.32 |
| Cerebrovascular accident or stroke | 127 (0.8) | 12 (0.6) | 44 (0.8) | 48 (0.9) | 22 (0.9) | 1 (0.3) | 0.57 |
| Severe arrhythmia | 1372 (8.5) | 161 (8.5) | 521 (9.0) | 484 (8.6) | 180 (7.5) | 26 (6.6) | 0.17 |
| Length of stay, day | 10 (7-13) | 10 (7-14) | 10 (7-13) | 10 (7-13) | 9 (7-13) | 10 (7-13) | 0.10 |
| **Medication at discharge** |  |  |  |  |  |  |  |
| Aspirin | 15066 (99.2) | 1726 (98.3) | 5440 (99.2) | 5266 (99.3) | 2249 (99.3) | 385 (99.5) | <0.0001 |
| Clopidogrel | 14868 (98.0) | 1701 (97.1) | 5365 (97.9) | 5202 (98.2) | 2218 (98.1) | 382 (98.7) | 0.08 |
| Statin | 14993 (98.7) | 1709 (97.5) | 5415 (98.8) | 5249 (99.0) | 2236 (98.8) | 384 (99.2) | <0.0001 |
| β-blocker | 11725 (77.3) | 1319 (75.2) | 4170 (76.1) | 4109 (77.6) | 1805 (80.0) | 322 (83.6) | <0.0001 |
| ACEI/ARB | 10135 (66.8) | 1014 (57.9) | 3479 (63.5) | 3652 (69.0) | 1689 (74.8) | 301 (78.2) | <0.0001 |

SMuRF=standard modifiable cardiovascular risk factor; ACEI=angiotensin-converting enzyme inhibitor; ARB=angiotensin receptor blocker; LVEF=left ventricular ejection fraction; PCI, percutaneous coronary intervention; CABG, coronary artery bypass graft.

**Table S3. Adjusted Proportions of Evidence-based Medication Use among Patients with First ST-segment Elevation Myocardial Infarction by Number of Cardiovascular Risk Factors**

|  | **Without SMuRFs** | **1-2 SMuRFs** | **3-4 SMuRFs** | **P_trend_** |
| --- | --- | --- | --- | --- |
| **Medication during hospitalization** |  |  |  |  |
| Aspirin | 97.2 (95.2-98.4) | 96.7 (94.8-97.9) | 97.2 (95.4-98.3) | 0.9065 |
| P_2_Y_12_-receptor inhibitor | 96.4 (93.8-97.9) | 97.5 (95.9-98.5) | 97.7 (96.0-98.6) | 0.0286 |
| Statin | 94.6 (91.6-96.6) | 95.3 (93.2-96.8) | 95.1 (92.6-96.8) | 0.6566 |
| β-blocker | 64.4 (59.1-69.4) | 63.3 (58.6-67.8) | 64.0 (59.1-68.7) | 0.9333 |
| ACEI/ARB | 55.6 (50.3-60.8) | 61.5 (56.9-65.9) | 67.9 (63.4-72.0) | <0.0001 |
| **Medication at discharge** |  |  |  |  |
| Aspirin | 99.3 (97.7-99.8) | 99.6 (98.8-99.9) | 99.6 (98.6-99.9) | 0.1218 |
| Clopidogrel | 98.6 (96.0-99.5) | 98.9 (96.9-99.6) | 99.1 (97.3-99.7) | 0.102 |
| Statin | 98.0 (94.9-99.2) | 98.8 (97.2-99.5) | 98.5 (96.3-99.4) | 0.4257 |
| β-blocker | 76.8 (71.5-81.3) | 76.5 (71.8-80.6) | 77.7 (73.0-81.9) | 0.4299 |
| ACEI/ARB | 71.0 (65.7-75.8) | 75.4 (71.0-79.3) | 80.7 (76.8-84.1) | <0.0001 |

Models were adjusted for age, sex, education level, hospital class, pre-hospital aspirin, systolic blood pressure, heart rate, creatinine, Killip class, cardiac arrest, onset-to-arrival time, anterior myocardial infarction, and reperfusion therapy.

**Table S4. Association of SMuRF status with in-hospital, 30-day, and 2-year mortality**

|  |  | **30-day mortality** | | **2-year mortality** | | **In-hospital mortality** | |
| --- | --- | --- | --- | --- | --- | --- | --- |
| **Factors** | **Reference** | **OR (95% CI)** | **P value** | **HR (95% CI)** | **P value** | **HR (95% CI)** | **P value** |
| **Unadjusted model** |  |  |  |  |  |  |  |
| **1-2 SMuRFs** | No SMuRFs | 0.73 (0.61-0.87) | 0.0006 | 0.75 (0.64-0.88) | 0.0004 | 0.79 (0.7-0.9) | 0.0005 |
| **3-4 SMuRFs** | No SMuRFs | 0.66 (0.53-0.83) | 0.0004 | 0.63 (0.52-0.78) | <0.0001 | 0.68 (0.58-0.8) | <0.0001 |
| **Adjusted model** |  |  |  |  |  |  |  |
| **1-2 SMuRFs** | No SMuRFs | 1.15 (0.96-1.39) | 0.14 | 1.19 (1.02-1.39) | 0.02 | 1.22 (0.95-1.55) | 0.12 |
| **3-4 SMuRFs** | No SMuRFs | 1.31 (1.02-1.68) | 0.03 | 1.39 (1.14-1.69) | 0.001 | 1.55 (1.14-2.12) | 0.006 |
| **Baseline characteristics** |  |  |  |  |  |  |  |
| Age |  | 1.04 (1.04-1.05) | <0.0001 | 1.05 (1.05-1.06) | <0.0001 | 1.06 (1.05-1.06) | <0.0001 |
| Male | Female | 0.66 (0.57-0.75) | <0.0001 | 0.76 (0.68-0.85) | <0.0001 | 0.59 (0.49-0.7) | <0.0001 |
| Education |  |  |  |  |  |  |  |
| Primary or secondary | Illiteracy | 1.02 (0.84-1.23) | 0.86 | 0.98 (0.84-1.14) | 0.78 | 0.95 (0.75-1.22) | 0.71 |
| High | Illiteracy | 0.88 (0.62-1.25) | 0.47 | 0.77 (0.58-1.02) | 0.07 | 0.78 (0.49-1.23) | 0.28 |
| Unknown | Illiteracy | 1.11 (0.89-1.37) | 0.36 | 1.01 (0.85-1.2) | 0.88 | 1.09 (0.82-1.43) | 0.56 |
| Hospital class |  |  |  |  |  |  |  |
| Prefecture-level | Province-level | 1.30 (1.09-1.54) | 0.003 | 1.29 (1.13-1.47) | 0.0002 | 1.32 (1.07-1.63) | 0.01 |
| County-level | Province-level | 1.79 (1.44-2.22) | <0.0001 | 1.64 (1.38-1.95) | <0.0001 | 1.89 (1.44-2.49) | <.0001 |
| BMI |  | 0.99 (0.97-1.01) | 0.15 | 0.99 (0.97-1) | 0.09 | 0.99 (0.96-1.01) | 0.29 |
| Pre-hospital aspirin - Yes | No | 0.96 (0.73-1.25) | 0.75 | 0.94 (0.77-1.16) | 0.57 | 1.06 (0.76-1.48) | 0.72 |
| Family history of CAD |  |  |  |  |  |  |  |
| Yes | No | 0.92 (0.57-1.47) | 0.71 | 0.73 (0.49-1.1) | 0.13 | 0.88 (0.49-1.58) | 0.67 |
| Unknown | No | 1.27 (1.08-1.50) | 0.004 | 1.17 (1.02-1.34) | 0.02 | 1.31 (1.05-1.62) | 0.01 |
| Prior history of stroke - Yes | No | 1.55 (1.30-1.84) | <0.0001 | 1.55 (1.35-1.78) | <0.0001 | 1.46 (1.16-1.84) | 0.001 |
| Prior history of COPD - Yes | No | 1.14 (0.84-1.56) | 0.41 | 1.22 (0.96-1.56) | 0.11 | 1.21 (0.78-1.87) | 0.39 |
| **Presenting characteristics** |  |  |  |  |  |  |  |
| Systolic blood pressure (per 10 mmHg) |  | 0.89 (0.87-0.92) | <0.0001 | 0.92 (0.9-0.94) | <0.0001 | 0.86 (0.83-0.89) | <.0001 |
| Creatinine (per 10 mmHg) |  | 1.03 (1.02-1.04) | <0.0001 | 1.03 (1.02-1.03) | <0.0001 | 1.04 (1.03-1.06) | <.0001 |
| Heart rate (per 10 beats/min) |  | 1.18 (1.14-1.21) | <0.0001 | 1.16 (1.14-1.19) | <0.0001 | 1.23 (1.18-1.28) | <.0001 |
| Killip class |  |  |  |  |  |  |  |
| II | I | 1.38 (1.17-1.64) | 0.0002 | 1.38 (1.21-1.57) | <0.0001 | 1.33 (1.07-1.64) | 0.009 |
| III | I | 1.78 (1.42-2.24) | <0.0001 | 1.84 (1.54-2.21) | <0.0001 | 1.92 (1.42-2.58) | <.0001 |
| IV | I | 2.56 (2.09-3.15) | <0.0001 | 2.51 (2.11-2.98) | <0.0001 | 3.64 (2.78-4.78) | <.0001 |
| Cardiac arrest - Yes | No | 2.06 (1.47-2.89) | <0.0001 | 1.69 (1.24-2.31) | 0.001 | 2.38 (1.48-3.85) | 0.0004 |
| Anterior myocardial infarction - Yes | No | 1.44 (1.25-1.66) | <0.0001 | 1.37 (1.23-1.53) | <0.0001 | 1.74 (1.45-2.08) | <.0001 |
| Onset-to-arrival time |  |  |  |  |  |  |  |
| 3-12 h | <3 h | 1.25 (1.03-1.51) | 0.0209 | 1.24 (1.07-1.43) | 0.005 | 1.27 (1.01-1.61) | 0.04 |
| 12-24 h | <3 h | 1.36 (1.07-1.74) | 0.01 | 1.23 (1.01-1.49) | 0.04 | 1.34 (0.98-1.83) | 0.06 |
| 1-7 d | <3 h | 1.01 (0.81-1.25) | 0.95 | 0.96 (0.81-1.14) | 0.67 | 0.99 (0.75-1.3) | 0.93 |
| **In-hospital management** |  |  |  |  |  |  |  |
| Reperfusion therapy |  |  |  |  |  |  |  |
| Thrombolysis | No reperfusion | 0.98 (0.77-1.24) | 0.87 | 0.92 (0.76-1.11) | 0.39 | 1.15 (0.86-1.54) | 0.34 |
| Primary PCI | No reperfusion | 0.57 (0.48-0.69) | <0.0001 | 0.58 (0.5-0.66) | <0.0001 | 0.54 (0.43-0.67) | <.0001 |
| Aspirin – Yes | No | 0.71 (0.55-0.92) | 0.01 | 0.7 (0.56-0.86) | 0.001 | 0.6 (0.42-0.87) | 0.007 |
| P2Y12 – Yes | No | 0.91 (0.68-1.2) | 0.49 | 0.85 (0.68-1.07) | 0.16 | 0.78 (0.53-1.16) | 0.22 |
| Statin – Yes | No | 0.47 (0.37-0.59) | <0.0001 | 0.49 (0.4-0.6) | <0.0001 | 0.33 (0.24-0.46) | <.0001 |
| β-blocker – Yes | No | 0.53 (0.46-0.61) | <0.0001 | 0.63 (0.56-0.71) | <0.0001 | 0.47 (0.39-0.56) | <.0001 |
| ACEI/ARB – Yes | No | 0.61 (0.53-0.71) | <0.0001 | 0.69 (0.61-0.77) | <0.0001 | 0.51 (0.43-0.61) | <.0001 |

OR, odds ratio; CI, confidence interval; HR, hazard ratio; SMuRF, standard modifiable cardiovascular risk factor; PCI, percutaneous coronary intervention; ACEI/ARB, angiotensin-converting enzyme inhibitor or angiotensin receptor blocker.

**Table S5. Sensitivity analysis for the association of SMuRF status with all-cause mortality at 30 days**

|  | Without SMuRFs | 1-2 SMuRFs | 3-4 SMuRFs | P_trend_ |
| --- | --- | --- | --- | --- |
| **Main analysis** | | | | |
| Unadjusted (n=16,228) | 1 (Ref) | 0.75 (0.64-0.88) | 0.63 (0.52-0.78) | <0.0001 |
| Adjusted (n=14,319) | 1 (Ref) | 1.15 (0.95-1.39) | 1.31 (1.02-1.68) | 0.03 |
| **SA1: excluding patients without lipid and glucose measurements at admission** | | | | |
| Unadjusted (n=14,154) | 1 (Ref) | 0.81 (0.66-1.00) | 0.80 (0.63-1.03) | 0.13 |
| Adjusted (n=12,904) | 1 (Ref) | 1.15 (0.92-1.44) | 1.45 (1.10-1.91) | 0.009 |
| **SA2: removing patients** **occurring death with the first 24 hours** | | | | |
| Unadjusted (n=15,804) | 1 (Ref) | 0.86 (0.70-1.06) | 0.74 (0.57-0.96) | 0.02 |
| Adjusted (n=14,027) | 1 (Ref) | 1.19 (0.95-1.50) | 1.31 (0.97-1.76) | 0.08 |
| **SA3: excluding patients transferring out** | | | | |
| Unadjusted (n=15,403) | 1 (Ref) | 0.74 (0.63-0.87) | 0.62 (0.50-0.76) | <0.0001 |
| Adjusted (n=13,633) | 1 (Ref) | 1.13 (0.94-1.37) | 1.29 (1.00-1.65) | 0.048 |
| **SA3: excluding patients occurring death with the first 24 hours and transferring out** | | | | |
| Unadjusted (n=14,987) | 1 (Ref) | 0.83 (0.68-1.03) | 0.71 (0.54-0.92) | 0.01 |
| Adjusted (n=13,346) | 1 (Ref) | 1.18 (0.93-1.48) | 1.28 (0.94 -1.73) | 0.11 |

Models were adjusted for Model 3 was adjusted for age, sex, education, hospital levels, BMI, family history of CAD, prior history of stroke, prior history of COPD, pre-admission aspirin, onset-to-arrival time, pre-admission cardiac arrest, heart rate, systolic blood pressure, plasma creatinine, Killip class, anterior myocardial infarction, reperfusion strategies, and evidence-based medications (aspirin, P2Y12-receptor inhibitor, statin, ACEi/ARB, and β-blocker). HR, hazard ratio; CI, confidence interval; SMuRF, standard modifiable cardiovascular risk factor; CAD, coronary artery disease; COPD, chronic obstructive pulmonary disease; ACEI, angiotensin-converting enzyme inhibitor; ARB, angiotensin receptor blocker.
